# High-energy proton imaging for biomedical applications

**DOI:** 10.1038/srep27651

**Published:** 2016-06-10

**Authors:** M. Prall, M. Durante, T. Berger, B. Przybyla, C. Graeff, P. M. Lang, C. LaTessa, L. Shestov, P. Simoniello, C. Danly, F. Mariam, F. Merrill, P. Nedrow, C. Wilde, D. Varentsov

**Affiliations:** 1 GSI Helmholtzzentrum für Schwerionenforschung GmbH, Planckstraße 1, 64291 Darmstadt, Germany; 2 Technische Universität Darmstadt, Schlossgartenstraße 9, 64289 Darmstadt, Germany; 3 Deutsches Zentrum für Luft- und Raumfahrt (DLR), Lindner Höhe, 51147 Cologne, Germany; 4 Brookhaven National Laboratory, P. O. Box 5000, Upton, NY 11973-5000, USA; 5 Frankfurt Institute for Advanced Studies (FIAS), Ruth-Moufang-Straße 1, 60438 Frankfurt am Main, Germany; 6 Los Alamos National Laboratory, Los Alamos, NM 87545, USA

## Abstract

The charged particle community is looking for techniques exploiting proton interactions instead of X-ray absorption for creating images of human tissue. Due to multiple Coulomb scattering inside the measured object it has shown to be highly non-trivial to achieve sufficient spatial resolution. We present imaging of biological tissue with a proton microscope. This device relies on magnetic optics, distinguishing it from most published proton imaging methods. For these methods reducing the data acquisition time to a clinically acceptable level has turned out to be challenging. In a proton microscope, data acquisition and processing are much simpler. This device even allows imaging in real time. The primary medical application will be image guidance in proton radiosurgery. Proton images demonstrating the potential for this application are presented. Tomographic reconstructions are included to raise awareness of the possibility of high-resolution proton tomography using magneto-optics.

Recent research on medical proton imaging is mostly motivated by the need for particle range determination in hadron therapy. Typically an X-ray CT scan is performed prior to a particle therapy treatment. This scan is used for outlining structures, but also provides a map of stopping power used to derive the beam energies necessary to focus the dose on the target region. The conversion of X-ray absorption, encoded in the grey values of the CT (Hounsfield units) to stopping power provides an appreciable source of error. This source of a systematic error could be avoided by proton computed tomography (proton CT), which relies on proton interaction instead of X-ray absorption. For a long time, the practical applicability of proton imaging remained limited, mostly because of insufficient spatial resolution (a few mm) due to multiple Coulomb scattering (MCS) inside an imaged object: When a proton beam traverses matter, the protons are not only slowed down in interactions with atomic electrons, but they are also deflected from their initial direction in electromagnetic interactions with atomic nuclei. The angular spread of a proton beam leaving an object is the result of many such deflections. If the scattered protons fall on a screen behind the object, their spatial distribution is very nearly Gaussian^1^. This makes proton images blurry unless a more sophisticated approach is chosen. Single particle tracking is one way to compensate blurring induced by MCS and current research has become focused on this method. We present an alternative method relying on magnetic optics to compensate for the image blur induced by MCS. This method even employs MCS for enhancing the image contrast. Both, single particle approaches and our method produce 2-dimensional images of an object. These can be combined to derive tomographic reconstructions of an object.

Yet, the primary motivation for the present investigation is a different one: A novel treatment modality has recently been proposed in the literature^2^. Relativistic proton beams are cross-fired through a region of diseased tissue e.g. in the brain. Tissue is sterilized at the dose maximum where the beams cross. Information on the traversed region is contained in the outgoing beam. This beam is used for online monitoring of the dose application process or even to implement an aim-and-shoot approach (Image Guided Stereotactic proton Radiosurgery, IGSpRS).

The PM can be used in combination with relativistic proton beams (energies around or above 1 GeV) making possible IGSpRS. This will be a research topic at the future Facility for Antiproton and Ion Research (FAIR, Darmstadt, Germany)^3^. The results presented in this article can be seen as the first step towards a realization of this technique. Tomographic reconstructions are included in order to bring wider awareness to the possibility of high-resolution proton computed tomography relying on magneto-optics. This approach is not widely known in the Medical Physics community and avoids some disadvantages of other methods.

For both applications, IGSpRS and proton tomography, the image resolution has to be better than 1 mm. As summarized below, quite complex methods have been invented to address this challenge. No proton imaging system has made it to the clinic yet.

The benefits of the PM can be summarized as follows: 1) high image resolution (about 0.2 mm for head sized objects), 2) the complete field of view can be measured with one short (e.g. 50 ns) proton spill, 3) short data acquisition time (in the order of 1 millisecond, including detector readout for a 2D image), and 4) simple data processing. Especially the combination of points 1) and 3) has proven to be difficult in the setups under development for proton computed tomography. In principle, the electronics of the setup use for the present investigation consists of a commercially availably CCD camera only.

This article is organized as follows: First we give a historical overview illustrating the challenge of producing high-resolution images using protons as probing particles. It will become clear that the combination of the above-mentioned benefits is unique. Afterwards the proton microscope is explained and experimental results are presented. The article concludes with a discussion and evaluates the role of the PM in medical research and its potential for future clinical applications.

## Background

The following mini-review is not essential for the understanding of the method we used, but helps to appreciate its benefits. The reader might skip this part and proceed with the explanation of the instrument in the following Section.

Proton imaging experiments of the last about 50 years were based on 1) particle attenuation, 2) nuclear scattering and 3) particle tracking combined with energy loss. Particle attenuation and energy loss setups work with non-relativistic energies. None of these approaches has made it to clinical trials yet.

Experimental attempts in proton radiography started with Koehler *et al*.^4^, who chose the particle attenuation approach using a scattered beam with E = 137 MeV. Proton flux attenuation was measured with a radiosensitive film placed downstream of the imaged object. The potential for tumor^5^ and stroke detection^6^ was demonstrated with brain specimens. Yet, MCS limited resolution to a few millimeters, which is too low, both for image guidance and for proton tomography.

In 1982 a group working at the Los Alamos National Laboratory (LANL, Los Alamos, NM, US) already produced pCT images of human specimens^7^ with a setup relying on a residual range measurement. A proton beam was scanned across the sample in a water tank, and the coordinates of the protons exiting the target were measured with a multi wire proportional chamber placed immediately before a range telescope. With this setup, the site of the myocardial infarction in a human heart was resolved. Even though the setup itself (object in a water tank) was not suitable for the clinic, the authors concluded that pCT could be a valuable tool for treatment planning, i.e. for the determination of stopping power in particle therapy.

It has been shown that in an instrument based on an energy measurement of the outgoing protons similar to the experiment just described, the blurring induced by multiple Coulomb scattering can be compensated to the sub-mm level if the individual proton trajectories are measured^8^,^9^. Recently this approach has become mainstream^10^. Most experimental setups aiming at clinical proton tomography employ tracking detectors in front of and behind the measured object. A detector following the second tracker is used to determine the particle energy after the passage of the sample.

With such a tracking system, an image of a dog’s head was produced at Paul Scherrer Institut (PSI, Villigen, Switzerland) with an E = 214 MeV proton beam under general anesthesia in a clinical environment in 2004 11. The bony anatomy was clearly resolved. After calibration with a water phantom, an uncertainty for proton range of about 0.6 mm was found. Spatial resolution was better than about 0.16 mm. The dose required for the imaging process was 0.03 mGy. Yet, the data acquisition time for a single 2D image was about 20 seconds. A complete tomographic scan would have required hours, which is clinically unacceptable (e.g. 2 hours with 360 projection angles).

Tracking systems are developed with a clinical application in mind^10^, they are relatively compact, they can be fitted into a treatment room and they can be rotated around a patient. The advantage of the PM is that its data acquisition time is much shorter. An image is available after a delay, which is in the order of a millisecond.

In tracking setups^12^ about 100 individual protons trajectories have to be accumulated per 1 mm^3^ of the scanned object^12^. In case of a typical medical application, the scanned volume will be in the order of 10^8^ mm^3^. For a clinically acceptable measurement time of about 10 seconds, a readout rate of 10 MHz (10^7^ trajectories/second) becomes necessary^13^. This requirement has proven to be challenging. The readout rate in many recent experiments ranges from 10–20 kHz^14^ to 1–2 MHz and various collaborations are investing considerable research into detector design^10^,^15^,^16^. Yet, readout rate is still a limiting factor. Special computer hardware might also be necessary to keep the reconstruction process within a time acceptable from the clinical point of view^17^.

There are also alternative approaches: Recent articles from the Heidelberg Ion Beam Therapy Center (HIT, Heidelberg, Germany) report the feasibility of imaging using carbon ions as probe. In that case, the MCS is significantly reduced due to the large projectile mass. A spatial resolution of less than one millimeter is achieved without single particle tracking. A flat panel detector^18^ and an IC stack^19^ were used to determine the stopping power in test samples.

We also mention the work of the Proton Radiography, Verification and Dosimetry Applications (PRaVDA) consortium. This collaboration is currently developing a novel silicon tracker for proton imaging and dosimetry^20^. This system might deliver results comparable to those of the PM in the near future. It might therefore also be a candidate for IGSpRS.

In the late 1970 s, Charpak’s group investigated the possibility of using protons having undergone nuclear interaction and scattered to large angles (larger than 19°) in order to obtain three-dimensional information of an object^21^,^22^,^23^. This experiment also relied on tracking detectors. Complexity of data acquisition prevented millimeter resolution in realistic objects. Published images of a canine head are so washed-out that they are illegible for non-experts^22^.

## The proton microscope

**Figure 1:**
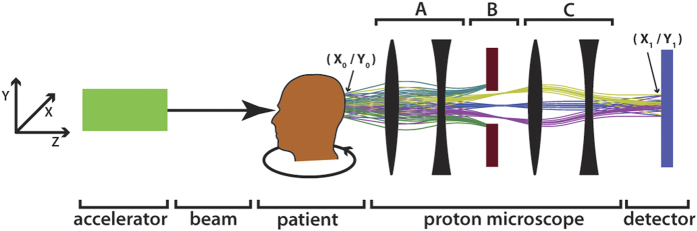
Operating principle of a proton microscope. An E = 800 MeV proton beam with a FWHM width of a few cm traverses an object and exits at the coordinates (X_0_,Y_0_). A first set of magnetic lenses (**a**) transforms the scattering angle to a lateral offset from the beam axis. The last set of magnetic lenses (**c**) reverses the effect of (**a**). In this way, a point-to point mapping between the exit coordinates (X_0_,Y_0_) and the coordinates at the detector (X_1_,Y_1_) is achieved. The collimator (**b**) removes protons having gained a scattering angle above a given limit after passing through a certain areal density in the object.

A PM (cf. Fig. 1), the instrument, which was used for the present investigation is conceptually different from the above-mentioned setups. It basically consists of an arrangement of four magnetic quadrupole lenses followed by a counting detector. The idea dates back to the mid-90 s^24^. In a PM, two-dimensional images with sub-mm resolution are created at the focal plane of a magnetic quadrupole lens, on a detector (cf. Fig. 1). The magnetic lens system a-c provides point-to-point proton imaging between the coordinates at the patient (*x*_*0*_*, y*_*0*_) and at the detector (*x*_*1*_*, y*_*1*_). This is an important feature. No electronics or data processing is required for compensating the blurring induced by MCS. The areal density of the traversed object is encoded in the proton flux. Due to MCS, protons leave the imaged object under scattering angles *θ* with respect to their initial direction. These angles *θ* roughly follow a Gaussian distribution with a width of a few milliradians^25^. In a PM a first set of magnetic lenses (a) maps the angle *θ* into radial distance from the beam axis at the position of the collimator (b): the larger *θ*, the larger is the distance from the beam axis. The collimator (b) removes protons with a scattering angle larger than *θ*_*c*_ given by its acceptance. A second set of magnetic lenses (c) placed after the collimator reverses the effect of the upstream lenses (a) and thus reforms the image. The thicker the measured object is, the more protons are scattered to large angles and the fewer reach the detector. Also this is achieved without any electronics or complex data processing steps, but with a static magnetic field and mechanical components.

The number of protons reaching the detector can be described with an analytical expression: The lateral scattering of a charged particle with atomic number *z* and mass number *A* is roughly proportional to *z*/*A β*^2^, where *β* is the ratio between the particle velocity *v* and the speed of light *c*. In our experiment, selecting an E = 800 MeV proton beam^2^ ensures that the lateral scattering inside the object remains below one millimeter. The standard deviation *θ*_0_ of the Gaussian describing the scattering angles of the outgoing protons is given by Highland’s formula^25^. It can be approximated as 
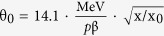
, where *x*_0_ is the radiation length, *p* is the proton momentum and *x* is the areal density of the measured object. In our experiment *θ*_0_ is in the order of a few milliradians. Additionally, nuclear interactions lead to a proton beam attenuation proportional to exp(*−d/λ*), where *λ* is the nuclear interaction length e.g. in a patient. Combining nuclear interactions and the scattering angle, the collimation leads to a transmission probability *T*(*d*):


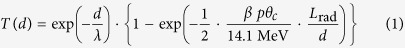


The function *T*(*d*) is strictly decreasing. Furthermore, matching magnets between the imaged object and the accelerator allow a correction of the most significant chromatic aberration term of the magnetic imaging system, hence to increase the spatial resolution of the microscope^26^ and to control the proton beam dimensions at the object.

It is a useful feature of this instrument, that one can illuminate the entire sample with a single scattered proton beam. For this purpose, tantalum plates^26^ were inserted in the primary beam to set its width in the object plane to FWHM = 3 cm and FWHM = 10 cm. Typically, 50 ns long pulses consisting of about 10^9^ protons were used to generate proton radiographs. The detector system consisted of a cesium-iodide scintillator whose output was collected by a conventional CCD camera. The light produced by the cesium iodide crystal was directed to a lens through two nearly 90° mirror reflections. The CCD was gated to collect light about 1 μs after beam arrival on the scintillator. This delay minimizes the background signal that results from secondary particles undergoing nuclear interactions within the silicon in the CCD camera, commonly referred to as “stars”^27^. These events result in a large amount of energy deposited in a single pixel, which then saturates. Whereas data acquisition for a single radiograph can take several seconds with proton tracking based setups^11^, data acquisition time with a PM is dominated by the CCD readout, which is in the order of a millisecond. This allows to visualize medical objects in real-time.

**Figure 2:**
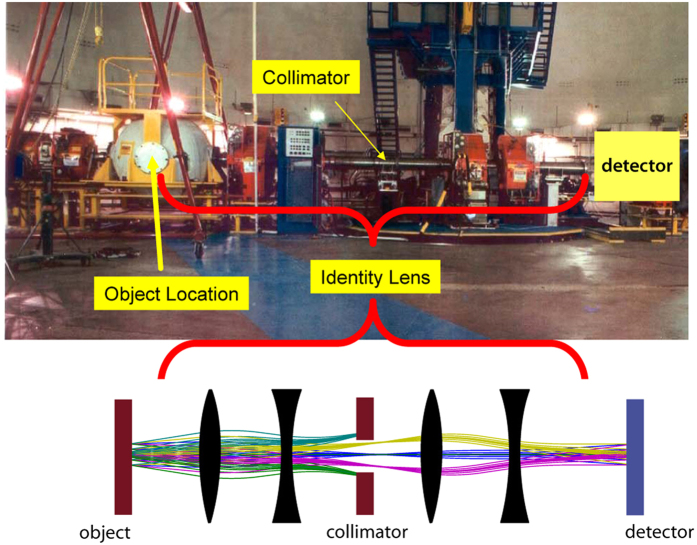
View of the proton microscope at the pRad facility at LANL. The spherical vessel on the left was removed for the experiments presented here. It was replaced by a rotary stage and the objects were irradiated outside the vacuum. Photography courtesy of Los Alamos National Laboratory.

To date, only a handful of PMs exist^28^. The pRad facility at Los Alamos National Laboratory (LANL, New Mexico, USA)^27^,^28^ was used for the present investigation (cf Fig. 2). This instrument is routinely employed for the study of explosive driven dynamic events^28^.

In this case, the explosion is contained in a steel vessel (left), which was removed for the present study. An automated manipulator for linear and rotary motion was set up at this location. All samples were moved e.g. for pCT measurements by this manipulator and acted as a sample holder at atmospheric conditions. A spatial resolution of 30 μm (far more than needed for medical applications) has already been demonstrated with the recently commissioned PM PRIOR-I at GSI Helmholtzzentrum für Schwerionenforschung (GSI, Darmstadt, Germany)^29^. A PM can also be used to magnify structures by adjusting the magnetic lens system.

## Results

The study presented here was designed to deliver qualitative results to demonstrate the potential for imaging of biological objects. Images clearly showing the potential for image guiding in IGSpRS were produced. Tomographic reconstructions are included to raise awareness for magneto-optics. An anthropomorphic phantom was used to simulate radiography of a human head. Proton images of a mouse sample show fine details in bony anatomy proving the sub-mm resolution of the method.

Images of chicken samples demonstrate the capability to resolve substructure in bones and soft-tissue. Metal pieces in chicken samples simulate fiducial markers as used for image guidance in medical applications.

### Proton radiography of biological samples

**Figure 3:**
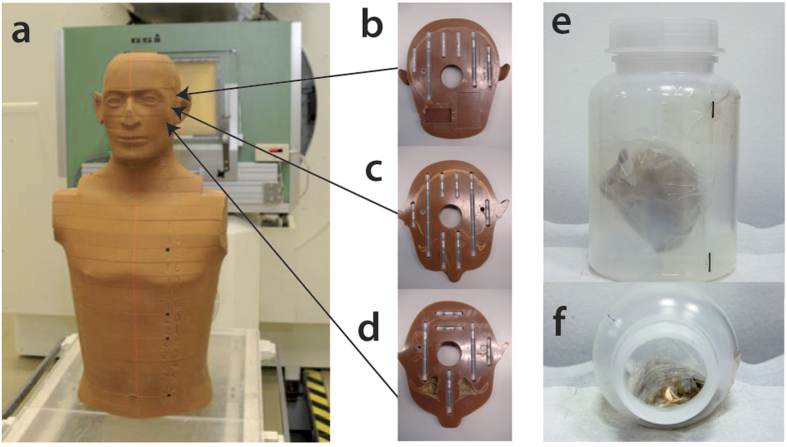
The main samples. The Matroshka phantom (**a**) was used to simulate radiography and tomography of a human head. The phantom is divided into 25 mm thick slices. Three of them (**b**,**c**) contain cavities which were filled with thermoluminescent detectors for dose measurements. Chicken samples (**e**,**f**) were fixed in plastic bottles. Metal pins were attached to the bottle to facilitate reconstruction of the axis of rotation for tomographic reconstructions.

Proton radiography on a human-like sample was tested irradiating the head and the torso of the anthropomorphic phantom Matroshka^30^,^31^. The head (cf. Fig. 3) has a maximum diameter of about 20 cm and is composed of several 25 mm thick slices.

**Figure 4:**
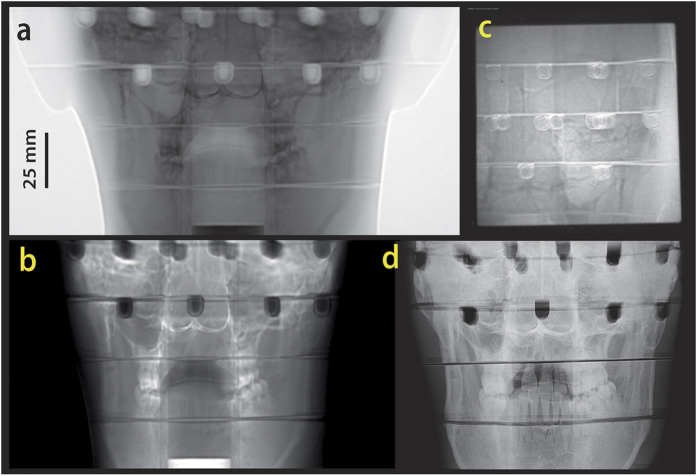
Images acquired from the radiography of the anthropomorphic phantom (Matroshka) head with a *θ*_*c*_ = 10 mrad collimator. (**a**) A merged radiograph. The grey values correspond to the number of transmitted protons. (**b**). Images converted to areal density (**c**). Unprocessed radiograph taken with 10 mGy imaging dose. (**d**) X-ray image (70 kV, about 5 μGy total dose).

A*θ*_*c*_ = 10 mrad collimator was used for the measurement. As the target was too wide for the system field of view, partial images were acquired and then merged (cf. Methods) in order to obtain a final radiograph for each angle. Geometric distortions caused by the detector system were compensated using proton radiographs of fiducial objects (cf. Methods). The numerically inverted transmission function was applied and the region outside the object was identified with zero density (cf. Fig. 4b). Using the same setup, images of a chicken sample (cf. Fig. 3) were collected.

Using thermoluminescent detectors placed inside the Matroshka head (cf. Fig. 3) we estimated a dose upper limit sufficient for obtaining an image in which the bony anatomy is clearly visible (cf. Fig. 4c). We emphasize that the setup was optimized for high-speed movies of explosive driven events^28^, the main application at LANL and not for low dose. The maximum point dose inside the head was found to be 10 mGy for such an image. In a typical X-ray, as shown in Fig. 4d, the imaging dose is about 5 μGy.

**Figure 5:**
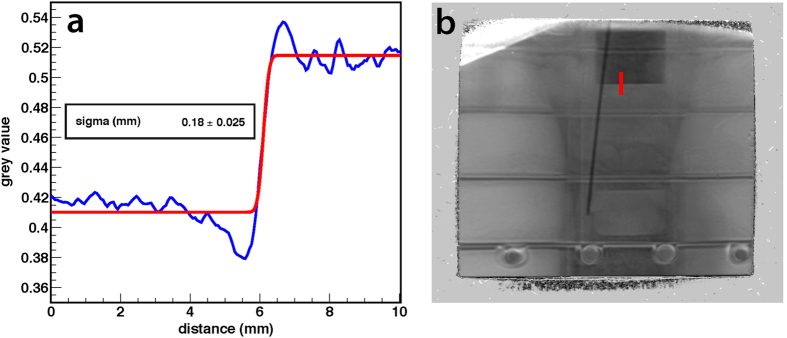
Image resolution in the chest region of the human phantom. Part (**a**) shows the grey values along the red line marked in (**b**). Inside the object protons are laterally scattered across the step of areal density. Data were collected with a *θ*_*c*_ = 10 mrad collimator.

In order to characterize experimentally the overall resolution determined by the combined effects of lateral scattering inside the phantom, the aberrations of the magnetic lens system and contributions from the detector system (cf. Methods), an aluminum cube was placed inside the phantom torso (cf. Fig. 5). The chest region was chosen for this measurement as it is the most homogeneous part of the phantom and thus allows an accurate examination of the aluminum box edges without having to consider other structures. The grey value transition between the box and its surrounding was fitted with an error function (stem function of the Gaussian distribution). The standard deviation calculated from the fit was σ = 0.18 mm (cf. Fig. 5). The under- and overshoot visible in the plot are systematic effects. At the interface between two regions with different densities, protons are laterally scattered, both from the dense into the less dense region and vice versa. In radiographs produced with a PM, this effect can cause fringes at a sharp boundary between two areas with distant densities (“limbing effect”). Whereas edges can be enhanced, this effect reduces the reconstruction accuracy in their vicinity. Similar measurements, performed with an E = 800 MeV proton beam using a PM setup at the Institute for Theoretical and Experimental Physics ITEP (Moscow, Russia) yield a similar spatial resolution of σ = 0.15 mm for the combination of PM and its detector system^32^.

### Proton tomography of biological and medical samples

Radiographic images were used to generate tomographic reconstructions with basic textbook methods. The Matroshka phantom, a chicken and a mouse sample were used for this purpose (cf. Methods). A series of 360 images collected in steps of 0.5° of the beam-synchronized rotary stage were acquired. Contrary to other published methods (cf. Section Background) data acquisition time was limited solely by the stepping speed of the rotary stage (0.5 Hz) and not by the instrument itself.

**Figure 6:**
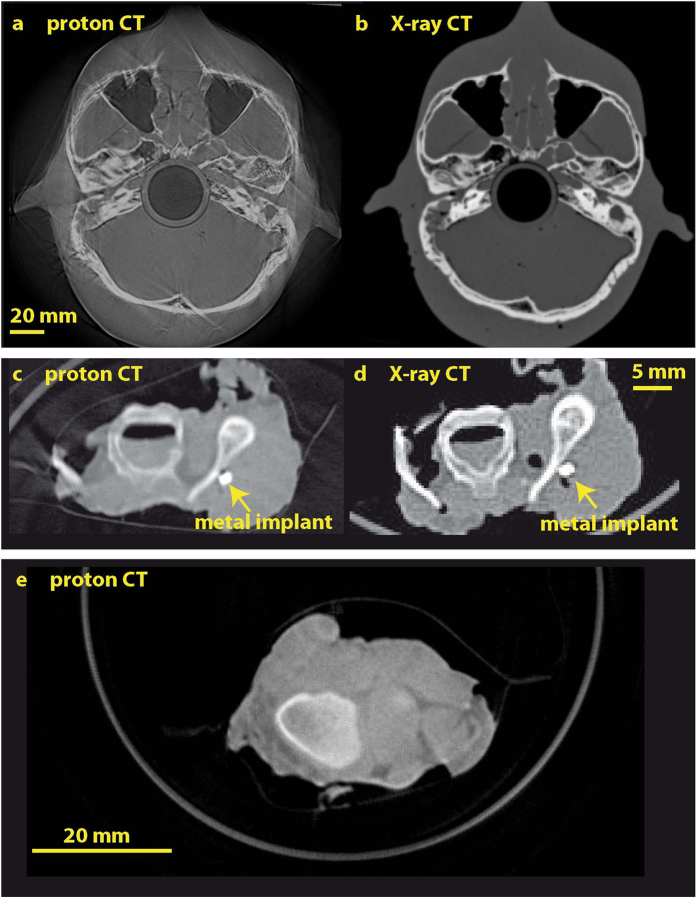
Comparison of proton and X-ray CT slices of the phantom head (**a**,**b**) and chicken sample (**c**,**d**). Bony anatomy and cavities are clearly visible in the proton CT of the phantom head (**a**). Streak artifacts were not suppressed. The proton CT of the chicken sample (**c**) shows bony anatomy and a metal implant placed close to a bone. The plastic in which the chicken part is wrapped is resolved as well. Image (**e**) shows structure in the muscle tissue of a chicken sample.

The filtered backprojection algorithm was used in combination with the Ram-Lak filter kernel^33^ to derive three-dimensional reconstructions. Proton CT slices clearly show bony anatomy (cf. Fig. 6a,c–e), metal implants (cf. Fig. 6c,d), and structure in the soft tissue (cf. Fig. 6e). The X-ray CT of the chicken sample was taken prior its shipment to LANL. This introduced some differences in the two images not due to the methodologies but rather in the sample structure itself. Proton CTs of the phantom skull (cf. Fig. 6a) contain streak artifacts indicating imperfect alignment and imperfect compensation of geometric distortions (cf. Methods) in the individual radiographs. Ring artifacts originating from correlations between the projection images dominated the mouse and chicken samples and thus have been suppressed (cf. Methods).

**Figure 7:**
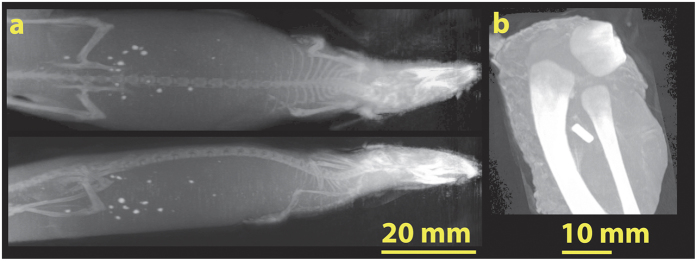
Maximum intensity projections (MIPs) generated from proton CTs acquired with the PM for a mouse (**a**) and chicken (**b**) sample. MIPs of the mouse show finest details such as vertebrae, ribs and finger bones, clearly underlining the high resolution of the method. The MIP of a chicken wing shows bones and metal fiducial. Data were taken with a *θ*_*c*_ = 5 mrad collimator.

Figure 7 shows maximum intensity projections of a mouse and a chicken sample derived from proton CTs. Such an image shows the maximum grey value along the path of parallel rays going though the proton CT. Especially the maximum intensity projection of the mouse underlines the high resolution achieved with the PM: ribs, vertebrae, shoulder blades and finger bones are clearly resolved.

**Figure 8:**
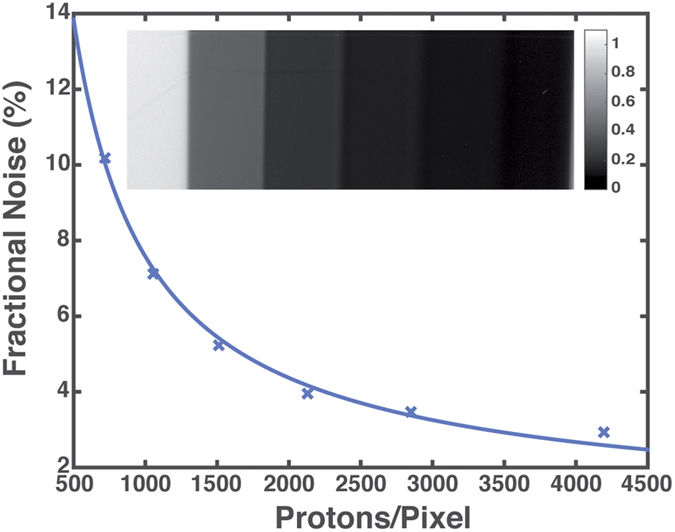
Fractional noise versus protons per pixel measured with an aluminum step wedge. The inset shows the fraction of protons transmitted through the PM for each step of the wedge.

An aluminum step wedge (cf. Fig. 8) was radiographed in order to characterize this system further. The noise was measured at each step through the step wedge, providing a measure of the noise as a function of proton intensity. This is also plotted as fractional noise versus number of protons per 50 micrometer pixel of the image. In the ideal system the noise will fall as 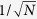, where *N* is the number of protons per pixel. The deviation from this ideal allows for an estimate of the noise floor and effective quantum efficiency of the full imaging system. This was estimated by varying the noise floor and effective quantum efficiency by fitting the noise versus signal data points (cf. Methods). The best fit noise floor is 30 protons and the effective quantum efficiency of the system is 50%, effectively using the protons used in these radiographs. It can also be observed that the transmission can be measured with this system at the few percent level on a length scale down to 50 micrometers. The transmission through larger features can be measured with higher accuracy. There also is another study^34^ in which densities were measured in an explosive driven experiment with the PM used. In this case, the measurement values reproduced the theoretical values with about 0.5 % accuracy.

## Discussion

The presented images clearly demonstrate that a PM can be used to produce images of biological objects like the human head with sub-mm resolution using protons having relativistic energies. The primary medical application will be a research project aiming at IGSpRS at Facility for Antiproton and Ion Research (FAIR, Darmstadt, Germany). Structures like the bony anatomy (cf. Fig. 4) or fiducials will be used for beam-guiding and patient setup.

A crucial feature at relativistic energies used for IGSpRS is that lateral scattering of the beam remains below 1 mm^2^,^35^. This makes it possible to work very close to life-critical regions. The IGSpRS project at FAIR can be seen as an improvement of proton beam treatment performed over a course of 30 years with about 1300 human patients at Petersburg Nuclear Physics Institute (PNPI, St. Petersburg, Russia)^35^. Various cancer and non-cancer diseases (mammary cancer, prostate cancer, pituitary adenoma, arterio-venous malformations) were treated in a single session with doses of up to 150 Gy^35^. Just as in the setup we propose, the beam direction was fixed and the patient was moved. No online imaging using the throughgoing beams was performed. In the Russian project, the irradiated volume was defined with respect to reference points on the head surface and the positioning procedure was performed with orthogonal X-ray. The combination with a PM allows online detection of setup-errors (as incorrect alignment or a misguided beam) during the treatment session. This is especially important when working close to life-critical structures; smaller safety margins can be used if necessary.

Also the short delay (order of 1 millisecond) of the PM data will be an important feature for treatments with very high doses. The irradiation can be interrupted if e.g. the patient coughs.

Based on the presented result we expect that a PM together with a similar detector as used for the present experiment is already suitable for IGSpRS. The imaging dose is of about 10 mGy is negligible compared to the high (up to 150 Gy) treatment doses. Some additional hardware will be installed to allow a workflow comparable to standard particle therapy (patient couch, room laser, X-ray system). For a future clinical application in a hospital, it is possible to design a specialized PM. The distance to the patient is a parameter, which can be chosen appropriately. A large distance (e.g. 5 m) is possible. For a possible initial pre-clinical phase, patients would have to travel to FAIR, a Nuclear Physics facility. This approach has already proven to be successful in the past. In a pilot project at the GSI (Darmstadt, Germany), the predecessor of FAIR, about 440 cancer patients were successfully treated with carbon beams between 1997 and 2008 36. The Heidelberg Ion Beam Therapy Center (HIT, Heidelberg, Germany)^37^,^38^ and the recently opened Marburg Ion Beam Therapy Center (MIT, Marburg, Germany) are the results of this pilot project.

It has already been emphasized in the literature that as it has become standard to perform routine imaging of the treatment beam in photon therapy; it will be the same in proton therapy too^10^. The benefit of proton imaging for patient positioning has been stressed long ago^39^. Also for such an application, a PM installed at a fixed position downstream of the patient and outside the treatment room could be used.

We emphasize that clinical proton CT is not the primary objective of the current study.

However none of the setups mentioned in the Background Section has made it to the clinic yet. The key idea is to employ magnetic optics for imaging purposes. As it should have become clear, this approach can dramatically reduce complexity of data acquisition and data processing compared to setups presented in the Background Section.

Several requirements on a pCT scanner, as mentioned in^17^, are already fulfilled with the PM used by us: 1) spatial resolution of <1 mm, 2) DAQ time <5 min, 3) reconstruction time <15 min, and 4) distance to the patient >10 cm. However, the PM employed is rather large and it was used with 800 MeV protons. Its size makes fitting on a gantry impractical and a gantry for these high-energy beams will probably not be implemented in the clinics either. For this reason, the proton tomographs produced should not be seen as direct feasibility study for a clinical application. The important idea is that magnetic optics can be used for proton CT. This approach avoids several disadvantages (complexity, measurement time). The results should be seen as incentive for further research investigating medical proton CT based on magnetic optics.

We conclude that we could successfully demonstrate a novel proton imaging method. It combines sub-mm resolution with low complexity, simple data processing and short data acquisition times. The primary application will be IGSpRS. This modality might become an important treatment for a subgroup of patients. It has already been emphasized in the literature that as it has become standard to perform routine imaging of the treatment beam in photon therapy it will be the same in proton therapy too^10^. There are various applications of proton imaging^40^. The benefit for patient positioning has been stressed long ago^39^. It should be clear that accurate patient positioning is mandatory in a high-dose treatment. The PM can also be used to produce high-resolution proton CT images. These can be acquired with unprecedented speed. Further research is necessary before a magneto-optical method can be considered for clinical proton CT. Various collaborations are trying to bring particle tracking setups to an applicable state. This method however cannot be used for IGSpRS. Dual energy X-ray CT might as well improve uncertainties in proton stopping power determination^41^,^42^,^43^ sufficiently to prevent the more complex proton CT in clinical reality.

## Methods

### Experimental workflow

The beam width was adjusted to FWHM ≈3 cm and FWHM ≈10 cm depending on the size of the measured object. Protons were counted by a detector system consisting of a 10 × 10 cm^2^ large CsI scintillator screen. The light output of the scintillator screen was collected via a mirror system by a triggered CCD camera as a proton pulse passed through the radiography system. In our case, the size of the scintillator screen limited the field of view, whereas the magnets aperture gave the physical limit.

The test samples were placed on a stage to rotate them in discrete angular steps for tomographic measurements. A proton pulse from the accelerator was sent though the sample between these steps, i.e. the rotary stage and the accelerator were synchronized. Two collimators with acceptance angles *θ*_*c*_ = 5 mrad and *θ*_*c*_ = 10 mrad were used.

### Image preprocessing

Beam and dark images were taken between the measurements of the test samples. The former were produced acquiring data without placing any sample in the object plane. Dark images were obtained reading out the detector signal with no beam and were subtracted from all raw images. Afterwards, the grey values of each picture were divided by the grey values of the image of the beam profile. Proton radiographs usually contained a few single saturated pixels caused by high-energy secondary particles, like scattered protons or neutrons, interacting within the camera chip. These outliers were removed. Before further processing, images were median filtered and resampled to 0.1 mm/pixel. Each radiograph was then divided by a beam picture to obtain a radiograph in which grey values correspond to areal density.

In case of the large Matroshka head, images were stitched together and geometric distortions coming from the mirror/lens part of the detector system had to be compensated. For this purpose, Lucite plates with evenly spaced tungsten rods were moved into the beam and radiographed at the beginning of a measurement period. Using the known geometry of this plate, deformation fields relating the measured with the actual geometry were derived and used to compensate geometric distortions in measured proton radiographs.

### Biological samples

The chicken samples (cf. Fig. 3) were fixed in 4% formaldehyde for 24 h, washed in phosphate buffered saline and then closed in an air tight plastic bag. Using adhesive tape, this bag was fixed in a plastic bottle. Metal pins were attached to the bottle to facilitate reconstruction of the axis of rotation for tomographic reconstructions.

The zebrafish was fixed in PFA 4% (by a ventral cut to let the fixative penetrate the interior cavities), for 6 hours. Then it was dehydrated in a graded ethanol series at room temperature: 70% (24 h), 95% (24 h), 100% (3 h). Incubation in xylene for 30 min concluded the dehydration process. The sample was then kept over-night in melted paraffin at 58 °C and embedded at room temperature. Before radiography, the paraffin embedding was thinned down to less than 0.5 mm.

### The Matroshka human phantom

The Matroshka phantom^30^,^31^ has been developed for space radiation experiments. It is designed to measure the dose distributions in critical organs, taking into account the mass distribution anisotropy of both the phantom itself and its shielding. Matroshka is an anthropomorphic upper torso (cf. Fig. 3) made of tissue equivalent polyurethane, which comprises a denatured human skeleton (RANDO®, The Phantom Laboratory, Salem, NY, USA). Its chemical composition consists of C, H, O, N and Sb with densities in a physiological range between 0.3 and 1.3 g·cm^−3^. It is cut horizontally into slices, each 25 mm in thickness, equipped with holders for radiation detectors. A bore hole is situated along the vertical axis of the whole phantom and it is usually filled (as for this experiment) with a plastic pipe to stabilize the relative positions of the slices.

The head and upper chest were used for the work presented here. As the Matroshka head is too wide to be imaged completely with the pRad setup, three separate images were acquired with a lateral shift of 7 cm. To achieve a vertical rotation axis, radiographs were adjusted by 1° and cropped by a few mm to avoid edge effects of the scintillator screen. Geometric distortions were compensated and the radiographs were stitched together. In the overlapping lateral regions (about 2 cm), grey values were linearly interpolated.

### Measurement of imaging dose

Cavities inside the Matroshka head (cf. Fig. 3) were filled with 61 thermoluminescent dosimeters. As these have their best dose response in the region of a few Gy, we accumulated dose with 80 radiographic measurements with a FWHM = 10 cm beam. The maximum point dose was about D_p_ = 10 mGy for a single (unprocessed) proton radiograph (one of 80 images) in which the bony anatomy is clearly visible (cf. Fig. 4c). No measurements with less than 10 mGy were performed. In a typical X-ray, as shown in Fig. 4d, the imaging dose is about 5 μGy. It has to be emphasized that in this work the setup was optimized for time-resolved radiography of explosive driven dynamic events and not for dose minimization.

### Ring artifact suppression

After reconstruction with the filtered backprojection algorithm, typical ring-shaped artifacts of a few pixels width disturbed the CT slices. These artifacts originate from correlated errors between pixel values of the radiographic projections. As the contrast from the thin objects itself is weak, the ring artifacts dominate. In order to suppress them, sinograms were preprocessed before filtered backprojection. There, the ring artifacts show up as vertical lines. The average row of each sinogram was computed. The corresponding grey values exhibited sharp spikes at the line positions. By subtracting a smoothed version of the average, low-frequency information was removed, while the high-frequency spikes were kept. The resulting profile was finally subtracted from each row of the sinogram before filtered backprojection.

### X-ray CT scanner

X-ray CT scans with a 1 mm slice spacing were acquired on a commercial PET/CT scanner (Biograph^TM^ TruePoint^TM^, Siemens Healthcare, Forchheim, Germany) at the Heidelberg Ion-beam Therapy Center (HIT, Heidelberg, Germany). The reconstruction slices of the proton and X-ray CTs are parallel.

### Fit function for the noise measurement

The fractional noise was fitted with the function given below. In this formula, σ_f_ is the root mean square noise floor in units of protons/pixel, DQE is the detected quantum efficiency, n is the number of protons per pixel.


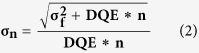


### Usage of animals

No live animals were used for the experiments.

## Additional Information

**How to cite this article**: Prall, M. *et al*. High-energy proton imaging for biomedical applications. *Sci. Rep*. **6**, 27651; doi: 10.1038/srep27651 (2016).
